# Research progress of bile biomarkers and their immunoregulatory role in biliary tract cancers

**DOI:** 10.3389/fimmu.2022.1049812

**Published:** 2022-10-28

**Authors:** Yun-cheng Li, Kang-shuai Li, Zeng-li Liu, Yong-chang Tang, Xiao-Qiang Hu, Xing-yong Li, An-da Shi, Li-ming Zhao, Li-Zhuang Shu, Shuo Lian, Zhang-di Yan, Shao-hui Huang, Guo-li Sheng, Yan Song, Yun-jia Liu, Fan Huan, Ming-hui Zhang, Zong-li Zhang

**Affiliations:** Department of General Surgery, Qilu Hospital, Cheeloo College of Medicine, Shandong University, Jinan, China

**Keywords:** biliary tract cancers, bile components, biomarkers, immune effect, liquid biopsy

## Abstract

Biliary tract cancers (BTCs), including cholangiocarcinoma and gallbladder carcinoma, originate from the biliary epithelium and have a poor prognosis. Surgery is the only choice for cure in the early stage of disease. However, most patients are diagnosed in the advanced stage and lose the chance for surgery. Early diagnosis could significantly improve the prognosis of patients. Bile has complex components and is in direct contact with biliary tract tumors. Bile components are closely related to the occurrence and development of biliary tract tumors and may be applied as biomarkers for BTCs. Meanwhile, arising evidence has confirmed the immunoregulatory role of bile components. In this review, we aim to summarize and discuss the relationship between bile components and biliary tract cancers and their ability as biomarkers for BTCs, highlighting the role of bile components in regulating immune response, and their promising application prospects.

## Introduction

Biliary tract cancers (BTCs) constitute a diverse group of malignancies emerging from the biliary epithelium, including cholangiocarcinoma and gallbladder carcinoma (1). According to its anatomical site, cholangiocarcinoma can be divided into intrahepatic cholangiocarcinoma(iCCA), perihilar cholangiocarcinoma(pCCA) and distal cholangiocarcinoma(dCCA) (2–4). iCCA, pCCA, and dCCA have distinct clinicopathologic characteristics in terms of epidemiology, molecular characteristics, clinical features, management, and outcomes (5). BTCs account for about 3% of all gastrointestinal tumors, and their incidence has increased in recent years (6). BTCs have an abysmal prognosis, with a five-year survival rate of about 10% (4). Radical resection is the only cure chance for BTCs. However, since patients are usually asymptomatic or only exhibit non-specific symptoms in the early stage, most patients are diagnosed at the advanced stage, missing the opportunity for radical surgery (7). Therefore, early diagnosis of BTCs is vital to improve patient prognosis.

The diagnosis of BTCs is based on a combination of imaging techniques, including ultrasound, CT, MRI, and tumor biopsy (5, 8).CT scanning is convenient and economical and is usually the first choice for diagnosing BTCs. MRI can provide high-resolution anatomical information about the biliary system, while MRCP is audio-visual (1). ERCP can directly display the biliary system and improve the accuracy of diagnosis. ERCP can also be used to confirm the diagnosis of cholangiocarcinoma by brushing cytology or biopsy (9). However, widely accepted biomarkers for diagnosing and dynamically monitoring the disease are still lacking. Currently, widely applied tumor markers, such as serum CA199 and CEA, have limited diagnostic value for BTCs (10). Tumor markers can be used for tumor screening, early detection, differential diagnosis and staging, prognosis judgment, efficacy monitoring, recurrence and metastasis monitoring, and guiding individualized treatment. The ideal tumor markers should have high sensitivity and specificity and reflect the tumor’s dynamic changes. The detection of tumor markers should be simple, fast, and accurate. Tumor markers have great application prospects in cancer. Thus, many researchers are working on searching for biomarkers used in the diagnosis, treatment response, and prognosis of BTCs (11–13). Tumor immunotherapy is a new and hot field. In addition, understanding the interaction between molecules and immune system is the premise of implementing immunotherapy.

Bile is produced by hepatocytes and secreted into the intestine through the biliary system. The compositions of bile are complex. Bile is composed mainly of bile acids, phospholipids, cholesterol, bilirubin, proteins, inorganic salts, etc. Proteins account for about 7% of the total bile compositions (14). Under the condition of disease, the ingredients of bile can be changed, especially in terms of BTCs, which is directly in touch with bile. Therefore, compared with blood or other body fluids, bile is an essential source for searching for tumor markers in the biliary system. Current advances in omics techniques have enabled researchers to discover new and valuable biomarkers in biological fluids (5). This review focuses on changes in bile composition and their interaction with the immune system. Changes in small molecules, proteins, ctDNA, miRNA, and extracellular vesicles have also been discussed.

### Small molecule biomarkers in bile

When tumors of the biliary system occur, small molecules in the bile are altered. Existing studies have focused on differences in bile acid molecules and lipids in bile. The production of bile acids in the liver is the body’s primary way of cholesterol metabolism. The primary bile acids, such as cholic and chenodeoxycholic acid, are synthesized from cholesterol in the liver, then secreted into the intestine to produce secondary bile acids through the action of intestinal bacteria. Primary and secondary bile acids are unconjugated and can be combined with glycine or taurine to form conjugated bile acids (15). The intestine can reabsorb more than 95% of all bile acids; the rest is excreted with the stool (16).

#### Bile acids

Bile acids can be divided into hydrophobic and hydrophilic bile acids according to their affinity for water. Hydrophobic and hydrophilic bile acids play opposite roles in tumor carcinogenesis. Hydrophobic bile acids, such as deoxycholic acids (DCA) and lithocholic acid (LCA), can promote tumorigenesis, whereas hydrophilic bile acids, such as ursodeoxycholic acid (UDCA), can prevent tumorigenesis (17–19). However, the relationship between the composition of bile acid in bile and the malignant transformation of biliary tract tumors has not been fully elucidated. Compared with patients with biliary stones or controls (liver transplant donors without biliary disease), patients with biliary system tumors had lower concentrations of total bile acids and lower ratios of DCA and LCA. For patients with bilirubin ≤ 2.0mg/dL, biliary tract tumor patients also showed lower total bile acid concentration and a lower percentage of DCA and LCA compared with the control group, suggesting its value in the early diagnosis of biliary tract tumors (20). A later study further confirmed the reduction in total bile acid concentrations. They also found an elevated ratio of primary bile acids and conjugated bile acids in CCA patients compared with BBD or PC, which is consistent with previous results (21, 22), indicating that conjugated bile acids can promote the growth of bile tract cancers. They further identified GCA as a positive biomarker and TUCDA as a negative biomarker for cholangiocarcinoma (23). Researchers have also found that compared with benign stenosis, the conjugated bile acids(C)/unconjugated bile acids(U) ratio of malignant stenosis patients is relatively low. As the C/U ratio decreases, the diagnosis rate of inflammation and tumors increases (24). The synthesis of bile acids is dynamically regulated and bile acid concentration can be a real-time and reliable indicator of liver function (25).However, the regulatory mechanisms of bile acid synthesis is complex and have not yet been fully elucidated. The results observed in those studies may reflect a partial imbalance in the process of bile acid during tumorigenesis.

In addition to digestive function, bile acids also participate in the regulation of immune function. Primary and secondary bile acids can inhibit the expression of pro-inflammatory cytokines from monocytes, macrophages, dendritic cells, and Kupffer cells (26). Bile acid metabolites affect the host immune response by directly regulating the balance of TH_17_ and Tregs (27). One LCA derivative, 3-oxolCa, can inhibit TH_17_ cell differentiation. Another LCA derivative, IsoalloLCA, can enhance Treg differentiation. Biliary primary and secondary bile acids can regulate the expression of RORγ^+^ regulatory T cells *via* bile acid nuclear receptor (28). Primary bile acids increase the expression of CXCL6, thereby causing aggregation of hepatic CXCR6^+^ NKT cells and producing anti-tumor effects through IFN-γ, while secondary bile acids have opposite effects (29). SIRT5 is a metabolic regulator involved in the occurrence and development of a variety of tumors and plays context-specific roles. A recent study found that the decreased expression of SITR5 in hepatocellular carcinoma patients leads to increased bile acid synthesis. Bile acids act as signaling molecules to stimulate their nuclear receptors and promote the polarization of M2-type macrophages, thereby creating an immunosuppressive tumor microenvironment and promoting tumor growth (30). It is reasonable to infer that bile acids play a role in the occurrence and development of biliary tract tumors by interacting with the immune system. In conclusion, bile acids play a complex role in immune regulation, and understanding the role of bile acids in immunity can promote the implementation of immunotherapy.

#### Lipids

Lipids include phospholipids, glycolipids, and cholesterol and their esters. Oxidized phospholipids(oxPLs) are essential in tumor cell apoptosis (31). Ten oxPLs were detected in bile from patients with diseases including CCA, PSC, PC, and BBD. Among them, ON-PC(1-palmitoyl-2(9-oxononanoyl)-sn-glycero-3-phosphatidylcholine) and S-PC(1-palmitoyl-2-succinoyl-sn-glycero-3-phosphatidylcholine) were significantly increased in CCA compared with other biliary stenosis patients. The sensitivity and specificity of ON-PC for distinguishing CCA from other biliary strictures were 85.7% and 80.3%, respectively. The sensitivity and specificity were increased to 100% and 83.3% by combining ON-PC and S-PC (32). The lipid peroxidation product 4- hydroxynonenal (4-HNE) was raised in gallbladder cancer patients compared with stones or controls. These findings indicate that lipids and their metabolites are also important bile markers for BTCs (33).

#### Other small metabolites

Metabolomics was applied to analyze low molecular metabolites in the body (10). The techniques used in metabolomics studies typically include Fourier transform infrared spectroscopy, gas chromatography/mass spectrometry(GC/MS), or lipid chromatography/mass spectrometry(LC/MS) (34). A new nuclear magnetic resonance spectra (NMR)-based metabolomics approach was also established to compare differences in the bile of patients with biliary tract cancer and benign biliary tract disease. A specific NMR spectroscopy can distinguish the two groups with 86% and 81% sensitivity and specificity, respectively (35). Lysophosphatidylcholine, phenylalanine, 2-octenoylcarnitine, and tryptophan levels in biliary tract cancer were decreased in patients of BTCs compared with benign diseases (36, 37).^1^H-MRS method was also applied to analyze the composition of bile. The levels of phosphatidylcholine and Taurine in bile were decreased in CCA patients compared to benign groups. The four regions in the final classifier (which represents phosphatidylcholine, bile acids, lipid, and cholesterol) can distinguish the two groups with a sensitivity and specificity of 88.9% and 87.1%, respectively (38). Another study examined volatile organic compounds in bile headspaces (gas above the sample) and found that several compounds (ethanol, acrylonitrile, acetonitrile, acetaldehyde, benzene, carbon disulfide, dimethyl sulfide, 2-propanolol) were decreased in patients with CCA compared with PSCs. A model was established based on the bile concentration of acrylonitrile, 3-methyl hexane, and benzene to distinguish patients with or without CCA. The sensitivity and specificity of the model are 90.5% and 72.7%, respectively (39).

Studies have focused on changes in other small molecules in the bile of patients with biliary tract cancer. The levels of glutathione (GSH), peroxide, ferrous iron (Fe^2+^), glutathione peroxidase (GP_X_), and farnesyl transferase/geranylgeranyltransferase type-1 subunit alpha (FNTA) in bile were investigated to illustrate the ferroptosis level of eCCA. The ability of these substances to distinguish between eCCA and common bile duct stones was further investigated. Compared with patients with common bile duct stones, the levels of these substances in bile are significantly reduced. Bile can be used as a biomarker source for diagnosing eCCA, especially for differentiating eCCA from benign bile duct stenosis (40). The lactate level in bile differs between the malignant disease group and non-malignant diseases or healthy people and may serve as a biomarker to distinguish benign people and malignancies (41). The content of ions and heavy metals in bile also changes in the diseased state. Compared with gallstones, patients with gallbladder cancer have decreased zinc levels and elevated copper and copper/zinc ratio (42). They tend to have higher concentrations of heavy metals such as chromium and cadmium (43).

### Protein biomarkers in the bile of CCA patients

Bile is believed to be the most relevant body fluid, which can closely indicate the pathological conditions of the biliary tract. Proteins are involved in tumor development (44–47). More and more data on protein biomarkers in bile is emerging. Serum CA199 and CEA are widely used markers for diagnosing biliary duct cancer, although the sensitivity and specificity are relatively low (48). Consequently, researchers have also studied the possibility of CA199 and CEA as bile biomarkers of CCA. The results indicate that bile CA199 and CEA are not valuable in distinguishing benign and malignant obstructive biliary tract disease (49). Thus, more potential bile markers with high sensitivity and specificity are urgently needed. Proteins in bile that may serve as tumor markers are summarized in **Table 1**.

**Table 1:** Bile proteins with potential diagnostic value for BTCs.

Source	Protein	Levels	Comparison	AUC	Sensitivity (%)	Specificity (%)	ref
bile	MUC5AC	Down	CCA (n=26) vs. benign biliary disease (n=20)	0.85	75	76.9	(50)
	WFA-Positive Mucin 1	Up	CCA (n=30) vs. benign duct disease (n=38)	0.86	90	76.3	(51)
	NGAL	Up	Malignant pancreatobiliary disease (n=16) vs benign biliary strictures (n=22) Pancreatobiliary cancers (n=22) vs. benign biliary strictures (n=18) CCA (n=30) vs. gallstone (n=36)	0.76 0.74 0.81	94 77.3 87	55 72.2 75	(52) (53) (54)
	CEACAM6	Up Up	CCA (n=41) vs benign disease (n=42) malignant (n=29) vs. benign biliary stenoses (n=12)	0.74 0.92	87.5 93	69.1 83	(55) (56)
	IGF-1	Up	Extrahepatic CCA (n=29) vs. benign biliary abnormalities or pancreatic cancers (n=44)	1			(57)
	VEGF	Down	CCA (n=9) vs. pancreatic cancer (n=5)	0.93	93.3	88.9	(58)
	Pancreatic elastase/amylase	Up	CCA (n=22) vs. gallstone patients (n=28)	0.88	81.8	89.3	(59)
	PKM2	Up	Malignant biliary stricture (n=17) vs. benign biliary stricture (n=29)	0.77	52.9	94.1	(60)
	Mac-2BP	Up	Biliary tract carcinoma (n=26) vs. benign biliary condition or PSC (n=49)	0.70	69	67	(61)
	Survivin	Up	CCA (n=55) vs. benign stricture (n=38)	0.78	67.3	80.9	(62)
	S100P	Up	CCA (n=14) vs. cholelithiasis (n=10)	0.86	92.9	70.0	(63)
	tFN	Up	carcinoma (n=21) vs. non-carcinoma (n=50)	0.93	88	95	(64)
	cFN	Up	carcinoma (n=21) vs. non-carcinoma (n=50)	0.97	91	98	(64)
	sB7-H3	Up	Malignant biliary disease (n=149) vs. benign biliary disease (n=174)	0.88	81.2	81.6	(65)
	sCD97	Up	ICC (n=7) vs. hepatolithiasis (n=10)	0.72	87.5	51.3	(66)
	LR11	Up	BTCs and PC (n=32) vs. benign biliary tract or pancreas related disease (n=40)	0.89	100	80	(67)
	Mcm5	Up	Malignant pancreatobiliary disease (n=44) vs. benign pancreatobiliary disease (n=16)	0.80	66	94	(68)
	HSP27	Up	CCA (n=10) vs. lithiasis (n=10)	0.86	90	90	(69)
	HSP70	Up	CCA (n=10) vs. lithiasis (n=10)	0.81	80	80	(69)

#### Mucin family

The mucin family is a group of glycosylated macromolecules. Some mucins are abnormally expressed in cancer cells and are involved in tumorigenesis and progression (70). Several mucin family members also exist in the bile and can serve as bile markers. MUC2 and MUC16 were the first identified mucin family members which were differentially expressed in bile between CCA and controls using proteomics methods (71), although the sensitivity and specificity of bile MUC2 and MUC16 in diagnosing CCA still need further validation. Previous studies have shown that intratumoral MUC4 and MUC5AC are associated with the diagnosis and prognosis of biliary tract tumors (72, 73). Therefore, whether bile MUC4 and MUC5AC are indicators of biliary tract tumors has attracted attention. Bile MUC4 was identified as a particular marker of BTCs (74). However, whether bile MUC5AC expression could distinguish benign and malignant biliary tract diseases is still controversial. A study reported that bile MUC5AC couldn’t distinguish benign from malignant biliary tract diseases (74). However, another study showed that bile MUC5AC could distinguish benign and malignant patients with higher diagnostic accuracy than serum MUC5AC (AUC=0.85 in bile vs. AUC=0.82 in serum). Evaluating MUC5AC in bile and serum simultaneously and based on the serum-to-bile ratio can achieve better diagnostic performance (AUC=0.97) (50).

As glycosylated macromolecules, studies have also focused on the performance of glyco-alteration of glycoproteins as a diagnostic marker of CCA. In a study, researchers have established a new sandwich ELISA system to detect the glyco-alteration of MUC1. The results revealed that bile *Wisteria floribunda* Agglutinin (WFA)-Positive Mucin 1 is a promising CCA biomarker, which can effectively distinguish CCA from benign biliary tract diseases with sensitivity and specificity of 90.0% and 76.3%, respectively and the AUC was 0.86 (51).

#### NGAL

Neutrophil gelatinase-associated lipocalin (NGAL), also called lipocalin-2 (LCN2), is a 25-kDa multifunctional protein released by activated neutrophils. NGAL plays a role in the onset and development of tumors (75). Zabron et al. compared the bile protein profiles of benign and malignant diseases. Biliary NGAL was the most valuable biomarker in distinguishing between benign and malignant groups. The area under the receiver-operating characteristic curve was 0.76 in differentiating malignant from benign pancreaticobiliary disease with sensitivity and specificity of 94% and 55%, respectively (52). Its diagnostic effect was validated in an independent cohort, and this capability is independent of CA199. The combination of bile NGAL and serum CA199 can improve the diagnostic accuracy for malignancy. Another study showed that bile NGAL levels could distinguish pancreaticobiliary cancer and benign biliary diseases with sensitivity and specificity of 73.3% and 72.2%, respectively. The area under the curve was 0.74 (53). In addition, Chiang et al. reported that biliary NGAL could differentiate CCA patients from gallstone patients with sensitivity and specificity of 87% and 75%, respectively. The AUC was 0.81. CCA patients with higher NGAL expression were also found to have poor overall survival (OS) of patients with CCA (54).

#### CEACAM6

Bile samples from patients having biliary stenosis caused by CCA or other malignant tumors were analyzed by the proteomics method (76). A total of 127 differentially expressed proteins were identified. Several of them have been reported to be associated with pancreatic cancer. Immunoblotting assays were performed to validate these markers’ expression, and significant CEACAM6 elevation in CCA has been identified. A subsequent study explored the role of bile CEACAM6 in diagnosing CCA. Bile CEACAM6 could discriminate cholangiocarcinoma from benign disease (AUC=0.738) (55). CEACAM6 was further screened and validated as a biomarker to distinguish malignant and benign bile duct stenosis with sensitivity and specificity of 93% and 83%, respectively. The AUC was 0.92. The combination of bile CEACAM6 and serum CA199 can improve the diagnostic accuracy for malignancy (AUC 0.96) (56).

#### Cytokines

CCA cells can secrete several cytokines and promote tumor progression by paracrine. Some of the cytokines secreted by CCA can also enter bile and may serve as biomarkers. Among them, IGF-1 and VEGF were the most studied. IGF-1 is a bioactive protein polypeptide, and it has been previously reported that serum IGF-1 levels are associated with prostate, breast, pancreatic, lung, and colorectal cancer (77, 78). VEGF promotes tumor angiogenesis, and the relationship between serum VEGF levels and various tumors has also been reported (79, 80). Expression levels of IGF-1 and VEGF in the bile and serum of extrahepatic cholangiocarcinoma, pancreatic cancer, and benign biliary abnormalities were measured to evaluate their role as a tumor marker (57). Biliary IGF-1 perfectly differentiated extrahepatic cholangiocarcinoma from benign biliary abnormalities or pancreatic cancer. The AUC was 1. Bile VEGF levels have no differentiating effect on biliary obstruction. Meanwhile, another study shows the level of bile VEGF could distinguish pancreatic patients from other etiologies of biliary stricture (58). Biliary VEGF-1 may help rule out distal common bile duct cancer (81).

Some cytokines related to chronic inflammation, such as IL-6 and TNF-a, play a role in the development of tumors by interacting with the immune system. Chronic inflammation-driven cytokines released from cholangiocytes, fibroblasts, or immune cells can promote the development of CCA (82).

#### Enzymes

BTCs are always complicated with an anomalous arrangement of pancreaticobiliary ducts and affect bile digestive enzymes, especially for pCCA and dCCA. Abnormal digestive enzymes and their relative product expression in bile may serve as biomarkers. One study used the quantitative proteomics method to compare bile proteins in six CCA patients with three different gross-appearance tumor types to identify possible biomarkers. They selected α-1- antitrypsin (AAT) for further validation. Fecal AAT, which can indirectly reflect bile AAT, can differentiate CCA from controls with a sensitivity and specificity of 80% and 75%, respectively. The AUC was 0.83 (83). Elevated amylase activity in bile is also associated with pancreaticobiliary reflux, a significant risk factor for biliary system tumors (84, 85). Therefore, high biliary amylase activity is associated with BTCs tumorigenesis (86). However, pancreaticobiliary reflux is affected by the location and extent of biliary tract obstruction. The amylase level in bile is not specific for diagnosing biliary tract cancers (87, 88). Compared with patients with gallbladder stones, the pancreatic elastase (PE) level in CCA patients is elevated. Biliary PE can distinguish patients with CCA from that with gallstones. The combined measurement of PE and amylase and the PE to amylase ratio can improve the accuracy of CCA diagnosis (AUC=0.0877) (59).

Dhar et al. (89) discussed the role of pyruvate kinase M2(PKM2) in the development of cholangiocarcinoma. They proposed that PKM2 can be used as a diagnostic marker of BTCs and is associated with prognosis. A subsequent study (60) showed that biliary PKM2 had low sensitivity (52.9%), but the specificity was high (94.1%) for the diagnosis of malignant and benign bile duct stenosis. The AUC was 0.77.

#### Mac2BP

In the first published bile proteomics, Mac-2-binding protein (Mac2BP) was identified as the potential biomarker for CCA. The value of Mac2BP as a diagnostic marker for cholangiocarcinoma was further validated in the following study (61). Bile Mac2BP can distinguish between malignant and benign biliary diseases with sensitivity and specificity of 69% and 67%, and the AUC was 0.70. The diagnostic effect is similar to biliary CA199 (AUC 0.69), and the combination of bile Mac2BP and CA19-9 can improve the accuracy of diagnosis (AUC 0.75).

#### Other bile proteins

Our group (62) applied ELISA to detect survivin and CA199 in bile. Biliary survivin could differentiate CCA and benign biliary obstruction with sensitivity and specificity of 67.27% and 80.85%, and the AUC was 0.78. Combining biliary survivin and CA199 could improve diagnostic accuracy (AUC=0.838).

Wang et al. (90) analyzed the proteins of gallbladder bile samples from gallbladder cancer, gallbladder adenoma, and chronic calculous cholecystitis patients using 2D LC-MS/MS. They proposed S100A8 as a potential biomarker for GBC and used immunohistochemical analysis to confirm it. Another study showed that S100P levels in bile were significantly elevated in patients with CCA, and bile S100P could distinguish between patients with CCA and choledocholithiasis with sensitivity and specificity of 92.9% and 70.0%, respectively (63).

Some other bile proteins including total fibronectin(tFN) and cellular fibronectin(cFN) (64), sB7-HB3 (65), sCD97 (66), LR11 (67), minichromosome maintenance protein 5(Mcm5) (68), HSP27, and HSP70 (69) was also reported to be bile biomarkers to distinguish benign and malignant biliary tract diseases with different sensitivity and specificity. However, these biomarkers still need further validation.

### ctDNA

The term liquid biopsy was first used to describe how the same diagnostic information can be obtained from blood samples as tissue samples. In oncology, the term broadly refers to the sampling and analysis of various biological fluids (91). Analytes include cell-free DNA(cfDNA), cell-free RNA(cfRNA), circulating tumor cells (CTCs), and extracellular vesicles (EVs) (91). The interaction between liquid biopsy components and anti-cancer immunity is a new area of research (92). CfDNA refers to fragmented DNA found in non-cellular components of blood, which are usually double-stranded fragments of about 150-200 base pairs in length (93). In cancer patients, the cfDNA released from tumor cells is called circulating tumor DNA (ctDNA) and constitutes part of the total cfDNA (94, 95).

Assessing the ctDNA in bile is a promising liquid biopsy method, especially for patients with insufficient tumor tissue to biopsy. However, whether the mutation profiles detected by ctDNA reliably reproduce the mutation profiles obtained by tumor biopsy remains a question (96). Bile contact with tumor cells directly, Therefore, detecting genetic alterations in bile may be more valuable than in plasma for biliary tract cancers. Several studies (97, 98) have demonstrated the feasibility of detecting genetic mutations of ctDNA in tumors of the biliary system, with a high compatibility rate with tissue biopsies. A recent study shows genetic mutations of ctDNA in bile are similar to those in tissues and are more effective than in plasma (97). Similar conclusions were reached in another study (98). cfDNA in bile samples is mainly long DNA fragments, significantly different from plasma samples. Targeted deep sequencing can reliably detect mutant variants in bile cfDNA. Tumor stage and location affect the sensitivity of mutation detection in bile cfDNA samples. In patients with GBC, the positive rate of mutation detected in bile ctDNA was 58.3%, which is higher than cytology. The mutation compliance rate between tissue DNA and bile ctDNA in GBC patients was 85.7%. KRAS and TP53 are frequent mutation genes in tumors of the biliary system. Several previous studies have evaluated the diagnostic effects of KRAS and P53 mutations in bile on tumors of the biliary system (99–102). Overall, the frequency of KRAS and P53 mutations in bile is too low to rely on these molecular markers to establish a reliable diagnosis.

It was initially thought that higher cfDNA might indicate tumor growth. However, many other diseases contribute to a similar increase. Thus, recent research has focused on epigenetic features of cfDNA, such as methylation (103). One study showed that hypermethylation of UCHL1 and RUNX3 in pancreaticobiliary fluid might be useful for diagnosing biliary duct cancers (104). INK4a/ARF’s promoter methylation status in bile might be useful for diagnosing benign and malignant biliary tract diseases (105). Shen et al. (106) established and validated a methylation panel in the bile, which can improve the sensitivity of detecting BTCs.

ctDNA can be used to diagnose biliary duct cancers at an early stage. Recently, a prospective study using ddPCR to detect the methylation status of four genes (CDO1, CNRIP1, SEPT9, VIM) in bile showed that detecting DNA methylation markers in bile allowed for more accurate and early detection of CCA patients in PSCs (107). Another prospective study suggests that next-generation sequencing (NGS) mutational analysis of bile cell-free DNA (cfDNA) in the initial stages of biliary stenosis can significantly improve the detection of malignant tumors. In addition, it can also test for the presence of mutations suitable for targeted therapy (108).

### miRNA and extracellular vesicles

miRNAs are small non-coding RNA molecules of 18-25 nucleotides in length, which can regulate gene expression by binding to corresponding mRNA sites (109). Extracellular miRNAs are stable and detectable in other body fluids (110). There are significant differences in miRNA concentration and composition in different diseases. Therefore, miRNA can be used as a biomarker to evaluate the body’s condition (111). Extracellular miRNAs are not homogenous groups and exist in various body fluids in different forms. Its origin and function are not fully understood. It is currently believed that some extracellular miRNAs are packaged in apoptotic bodies, shedding microvesicles, exosomes, or high-density lipoprotein (HDL) particles. Most extracellular miRNAs (90%-95%) bind to AGO proteins (110).Therefore, attention should be paid to the origin of the miRNAs when evaluating their roles as biomarkers.

Extracellular vesicles are all kinds of vesicles with membrane structures released by cells, and their contents include proteins, lipids, nucleic acids, and so on. According to the diameter and occurrence mode, it can be divided into exosomes, microvesicles, and apoptotic bodies (112, 113). Tumor-derived exosomes are likely to serve as potential cancer markers.

Shigehara et al. (114) compared the difference of miRNA in the whole bile between patients with malignant and benign biliary tract diseases for the first time and pointed out that miR-145 and miR-9 could be used as diagnostic markers of BTCs (**Table 2**), especially miR-9, which has higher diagnostic accuracy. A subsequent study showed that RNA from free-floating cells degrades rapidly. The bile processing process can produce unpredictable deviations. Therefore, they focused on extracellular vesicles in bile instead of the whole bile and established a miRNA panel based on extracellular vesicles in bile to diagnose CCA with a sensitivity and specificity of 67% and 96%, respectively (115). However, miRNA in extracellular vesicles only accounts for a small part of extracellular miRNA. Thus, one study suggested that extracellular vesicles were not a reliable miRNA source for CCA diagnosis. They recommend cell-free bile rather than whole bile or extracellular vesicles in bile to find potential miRNA markers (118). miRNA profiles in the bile of PSC, PSC with CCA, and CCA patients were different, among which miR-142, miR-640, miR-1537, and miR-3187 were significantly different in PSC and CCA patients (116) (**Table 2**). In another study, extracellular miR-30d-5p and miR-92a-3p were increased dramatically in the bile of CCA patients compared with benign biliary disease (BBD) patients (117) (**Table 2**). miR-30d-5p showed the best performance in distinguishing CCA and BBD patients. In addition, it has been demonstrated that high methylation of miR-1247, -200a, and -200b in bile may help determine benign and malignant biliary tract diseases (119).

**Table 2:** Bile miRNAs with potential diagnostic value for BTCs.

Source	miRNA	levels	Comparison	AUC	Sensitivity (%)	Specificity (%)	ref
bile	miR9 miR145	up	Malignant (n=9) vs. benign biliary disease (n=9)	0.98 0.98	88.9 77.8	100 100	(114)
	miR-191 miR-486-3p miR-1274b miR-16 miR-484	Up	CCA (n = 46) vs. Control (n = 50)	**/**	67	96	(115)
	miR412 miR640 miR1537 miR3189	up up up up	CCA/PSC (n=12) vs. PSC (n=52)	0.81 0.81 0.78 0.80	50 50 67 67	89 92 90 89	(116)
	miR-30d-5p MiR-92a-3p	up up	CCA (n=37) vs. BBD (n=48)	0.73 0.65	81.1 65.7	60.5 66.7	(117)

The extracellular vesicles can also be used to distinguish between patients with benign and malignant diseases. As aforementioned, an established miRNA panel (**Table 3**) in the extracellular vesicles can be used to diagnose CCA (115). Ge X et al. (120)compared the expression of lncRNAs in bile exosomes from CCA patients and biliary obstruction patients. They identified that two lncRNAs were significantly elevated in CCA, and the combined use of two lncRNAs showed better diagnostic value (**Table 3**). Besides, both of them were associated with CCA prognosis. Bile exosome circCCAC1 can distinguish CCA from benign hepatobiliary diseases (AUC=0.857) while tissue circCCAC1 is associated with the prognosis of patients (122). In addition to non-coding RNA, proteins in extracellular vesicles can also be used as markers for patients with CCA. Ikeda, C et al. (121)used EDEG to separate human bile EVs and analyzed proteins in extracellular vesicles for the first time. They found that Claudin-3 in bile EVs can distinguish between CCA and patients with bile duct stones with a sensitivity and specificity of 87.5% and 87.5%, respectively (**Table 3**). The concentration of EVs in bile may be relevant to diagnosing malignant biliary tract disease. One study showed that the concentration of EVs in bile can distinguish malignant from non-malignant CBD stenosis with 100% accuracy (123).

**Table 3:** Bile EVs with potential diagnostic value for BTCs.

EV source	Name	Biomarker type	Levels	Comparison	AUC	Sensitivity (%)	Specificity (%)	ref
bile	miR-191 miR-486-3p miR-1274b miR-16 miR-484	miRNA	Up	CCA (n = 46) vs. Control (n = 50)	**/**	67	96	(115)
	ENST00000588480.1* ENST00000517758.1*	lncRNA	Up	CCA (n = 35) vs. Control (n = 56)	0.71	82.9	58.9	(120)
	Claudin-3	Protein	Up	CCA (n=8) vs. bile duct stone (n=8)	0.95	87.5	87.5	(121)

*combined use

In addition to being a marker of disease, the potential therapeutic role of EVs in disease is gradually being discovered (124). EVs can target receptor cells, increase drug concentration in target tissues, and reduce drug toxicity and side effects. The outer membrane of the vesicle increases drug stability and prevents drug degradation. The study of vesicle-related functions has become a research hotspot and is expected to play a role in the early diagnosis and treatment of many diseases.

## Discussion

BTCs are a group of highly heterogeneous diseases with abysmal prognosis. Finding a method for early diagnosis of BTCs in a non-invasive or minimally invasive way and evaluating the treatment effect to improve the prognosis of patients with BTCs is promising. The search for tumor markers is a feasible approach. Many previous studies have focused on finding a substance in the blood, evaluating its relationship with BTCs, and determining whether it can be used as a tumor marker. Blood samples are readily available, but blood often reflects the body’s overall conditions and cannot be accurately localized to an organ or tissue. The components in the blood may interfere with each other and mask the characteristic information of the disease, especially in the early stages (125). Therefore, many studies have focused on finding tumor markers in other body fluids. Bile is directly exposed to BTCs, and tumors will release certain substances into bile. Thus, bile may contain tumor-specific information, an essential source for searching for tumor markers. The acquisition of bile is invasive, and it is difficult to obtain information about a healthy person’s bile (14). While in the disease state, some treatments such as ERCP, PTCD, and surgery become essential sources of bile without adding additional trauma to patients. Therefore, bile biomarkers research is more valuable. This study focused on bile and summarized the research progress of the relationship between bile components and BTCs.

Metabolomics is used to analyze low molecular metabolites in bile to determine the pathophysiology of the human. Bile acids and lipids are two kinds of small molecules that are most concerned at present. However, the results of different studies are inconsistent or even opposite. Many factors are involved, including pretreatment before bile testing, tumor anatomical site and methods of bile collection, individual differences in disease severity, and various other factors affecting the results. For the early diagnosis of BTCs, more attention was paid to whether bile composition changes in the absence of biliary obstruction (20). Bile acids and lipids are involved in the occurrence and development of BTCs, and the mechanism needs to be further studied.

Proteomics studies the composition and variation of proteins in cells, tissues, or living organisms. Clinical detection of disease is mainly based on protein analysis. Bile protein analysis is challenging to carry out because of the interference of many other substances (14). Thus, pretreatment is required before proteomic analysis. The proteome is a fluctuating description of the host’s response to disease. Many minor but significant changes may occur in the early stage of the disease (126).Therefore, combining multiple markers is more effective than single markers (127). Protein changes arise during the malignant transformation of healthy cells (128–130). Researchers currently focus on finding overexpressed proteins that flow into body fluids due to disease as disease biomarkers (131–133). Since bile is directly in contact with BTCs, tumor markers of BTC are more likely to be identified in bile. The use of mass spectrometry to analyze proteomic patterns in tissues and body fluids, combined with bioinformatics tools to distinguish patterns in normal, benign, or malignant disease states, namely, proteomic pattern diagnosis (134). Thus, artificial intelligence (AI) is required for processing high-throughput omics data.

Liquid biopsy is a hot field now, which can be used to detect cfDNA, cfRNA, CTCs, EVs, etc. (91). Compared with the traditional methods, it can comprehensively reflect the overall state of the tumor in a minimally invasive and fast way, and it applies to a wide range of people. Liquid biopsies are good supplements for patients who have difficulty obtaining biopsy tissues or have insufficient tissues for genetic testing. Liquid biopsies can be used to diagnose and guide the treatment of patients (135). The low ctDNA content of the samples used for liquid biopsies, especially in the early stages, poses a great challenge to the detection of ctDNA (135). ctDNA is currently the most widely used in clinical practice. ctDNA can be used as a tumor marker of BTCs by detecting the related mutation status of ctDNA (125). ctDNA in bile provides a comprehensive view of the tumor genome, reflecting DNA released from multiple tumor regions. It is dynamic. Thus, ctDNA in bile can monitor treatment response, drug-resistant, tumor heterogeneity, and detect residual disease after surgery. In conclusion, detecting ctDNA in bile is an auspicious method.

Multiple miRNAs have been found in the blood as markers of BTCs. However, miRNAs are rarely reported in bile. Extracellular miRNAs are heterogeneous and may play an essential role in the occurrence and development of BTCs (110). Extracellular miRNAs may act as tumor markers for BTCs and are associated with prognosis. EVs are a recently emerging class of molecules that play an essential role in cell-to-cell communication, with exosomes being the most mature (113). There are also growing studies of EVs in bile. EVs contain various substances such as proteins, nucleic acids, etc. These substances within EVs or the concentration of EVs in bile may play an essential role in the occurrence and development of BTCs and may act as biomarkers for BTCs. EVs can also be used in the treatment of diseases. Bile-specific exosomes may lead to better therapeutic outcomes. Bile enters the intestine through the biliary tract and may be helpful in the treatment of intestinal diseases (124).

In addition to traditional chemotherapy and targeted therapy, tumor immunotherapy is a revolutionary breakthrough. Instead of acting on tumor cells, it activates the body’s immune system to produce an anti-tumor effect. Understanding the mechanism of interaction between molecules and the immune system is immunotherapy’s premise. Anti-tumor immunotherapy is promising theoretically and ideologically. However, it still seems a long way to go.

### Future perspectives

Bile biomarkers are promising and meaningful in diagnosis and prognosis judgement of CCA. However, bile collection is invasive, and it is difficult to obtain normal bile from a healthy donor (14). Setting the control group to patients with benign diseases may lead to unpredictable biases. Instead, finding a non-invasive way, such as blood, feces, etc., to get an accurate diagnosis is preferred. However, this requires building a relationship between bile and the clinically readily available samples such as blood, urine, stool, etc. Besides, single bile biomarker is hard to reach a high specificity and a high sensitivity simultaneously. An accurate BTCs diagnosis involves combining data from multiple omics, including genomics, epigenomics, transcriptomics, proteomics, and metabolomics. The high-throughput multi-dimensional information, combined with AI technology, makes it possible to diagnose BTCs more accurately and improve the judgment of patient prognosis.

## Author contributions

Y-CL, K-SL, Z-LL, Y-CT, and X-QH collected the references. Y-CL and K-SL analyzed the references and wrote the paper. X-YL, A-DS, L-MZ, L-ZS, SL, Z-DY, S-HH, G-LS, YS, Y-JL, FH, and M-HZ read the paper and provided revising advice. Z-LZ contributed to study supervision and revised the manuscript. All authors contributed to the article and approved the submitted version.

## Funding

This work was supported by the National Natural Science Foundation of China (Grant No. 81900728, 82072676,82172791,82203766), Shandong Province Natural Science Foundation (Grant No. ZR2019MH008, ZR2020MH238, ZR2021QH079), Shandong Province Key R&D Program(Major Scientific Innovation Projects,2021CXGC011105), Shandong Medical and Health Technology Development Project (Grant No. 2018WSB20002), Clinical Research Foundation of Shandong University (Grant No. 2020SDUCRCA018), Key Research and Development Program of Shandong Province (Grant No. 2019GSF108254). The funders had no role in study design, data collection, analysis, interpretation, and manuscript writing.

## Conflict of interest

The authors declare that the research was conducted in the absence of any commercial or financial relationships that could be construed as a potential conflict of interest.

## Publisher’s note

All claims expressed in this article are solely those of the authors and do not necessarily represent those of their affiliated organizations, or those of the publisher, the editors and the reviewers. Any product that may be evaluated in this article, or claim that may be made by its manufacturer, is not guaranteed or endorsed by the publisher.

## Glossary

**Table:**

BTCs	biliary tract cancers
CCA	cholangiocarcinoma
iCCA	intrahepatic cholangiocarcinoma
pCCA	perihilar cholangiocarcinoma
dCCA	distal cholangiocarcinoma
eCCA	extrahepatic cholangiocarcinoma
MRI	magnetic resonance imaging
MRCP	magnetic resonance cholangiopancreatography
ERCP	endoscopic retrograde cholangio-pancreatography
DCA	deoxycholic acids
LCA	lithocholic acid
UDCA	ursodeoxycholic acid
BBD	benign biliary diseases
PC	pancreatic cancer
GCA	glycocholic acid
TUCDA	tauroursodeoxycholic acid
WFA-Positive Mucin 1	wisteria floribunda agglutinin-positive Mucin 1
NGAL	neutrophil gelatinase-associated lipocalin
PKM2	pyruvate kinase M2
Mac-2BP	Mac-2-binding protein
tFN	total fibronectin
cFN	cellular fibronectin
Mcm5	minichromosome maintenance protein 5
PSC	primary sclerosing cholangitis
CA199	carbohydrate antigen199
CEA	carcinoembryonic antigen
CEACAM6	carcinoembryonic antigen-related cell adhesion molecule 6
IGF-1	insulin-like growth factors -1
VEGF	vascular endothelial growth factors
sB7-H3	soluble B7-H3
sLR11	soluble LDL receptor relative with 11 ligand-binding repeats
HSP	heat shock proteins
miRNA	microRNA
lncRNA	long noncoding RNA
